# A history of visual acuity testing and optotypes

**DOI:** 10.1038/s41433-022-02180-6

**Published:** 2022-08-03

**Authors:** Paulus T. V. M. de Jong

**Affiliations:** 1 Department of Retinal Signal Processing, Netherlands Institute of Neuroscience, KNAW. Meibergdreef 47, 1105 BA Amsterdam, The Netherlands; 2 Department of Ophthalmology, AmsterdamUMC, Meibergdreef 9, 1105 AZ Amsterdam, The Netherlands

**Keywords:** Scientific community, Diagnosis

## Abstract

After going into the etymology of the word “optotype”, this article covers some tasks in ancient times that required good visual acuity (VA). Around 300 BCE, Euclid formulated the existence of a visual cone with a minimal visual angle at its tip. Trials to test VA appeared AD 1754. Around that time, texts were introduced by opticians in order to be able to prescribe more reliably. In the early nineteenth century, the need for VA tests in ophthalmology resulted in German and English test charts. Numerous variants emerged after the first edition of Snellen’s optotypes in 1862 in The Netherlands. However, 100 years later there was still no standard optotype to reliably test VA. Multidisciplinary approaches between ophthalmology, linguistics, psychology and psychophysics improved optotypes and VA testing, which led to the more reliable LogMAR charts. Recent advances in aids and therapies for the blind and severely visually handicapped, necessitate further development of new and standardized VA tests.

## Abstract (zh)

在深入研究“视力表”的词源后, 本文阐述了几个古代需要获得视力的一些重要事件节点。大约在公元300年, Euclid 提出了“视锥”的存在, 即反映在视角尖端的最小角度。视力检查出现于公元1754年。当时, 验光师为了获得更精准的验光处方引入了视力检查。在19世纪早期, 对于眼科视力检查的需求刺激了德语和英语视力表的产生。1862年, 第一版Snellen视力表在荷兰问世后, 大量不同的版本也随之涌现。然而, 100年后依然没有标准视力表能够可靠地检测视力。眼科学、语言学、心理学、心理物理学等多学科方法改进了视力表和视力检查, 这也促使了更为可靠的LogMAR视力表的产生。在盲人和严重视力障碍患者的辅助和治疗的最新进展中, 需要进一步开发新的标准化视力检查方法。

## Introduction

In various dictionaries, “Optotype” is defined as: *A type or letter of definite size used for testing acuteness of vision* [1]*, a type by which to test the eyesight* [2], or *test type* [3]. The latter is explained as *Printed letters of varying size, used in the testing of visual acuity; see also under chart. The test types are subdivided into those of Jaeger, Landolt, and Snellen* [3]. These definitions give limited guidance on what exactly an optotype is. The word “optotype” was described as originating from the German “Optotypus” [1] but Herman Snellen from Utrecht, The Netherlands, was the first to coin this word in 1875. It did not appear in the first edition of his “Lettertypen” [4] but 13 years later, he introduced “Optotypi” in his international test type edition in Latin [5]. It is uncertain how he invented this word. Presumably, he composed a neo-Latin word from the Greek words οπτος (optos, visible) and τυπος (tupos, model to be imitated) [6]. For the sake of clarity to the reader, I present here a working definition of optotype: An optotype is a visual aid to arrive at a *dependable and standardized* measure of visual acuity (VA) on arbitrary but commonly agreed grounds. These aids gradually changed from printed texts, letters, numerals or figures, to a variety of forms. They could be printed with high or low contrast in different colors, pasted on a transparent screen with lighting behind, projected on a (computer) screen, or made of metal or other material shown to the test person. In clinical ophthalmology, optotypes are used in two ways: to aid in finding the optimal lens to correct a refractive error of an eye and to determine its VA.

This paper will start with some notes on vision in ancient times followed by discussion on the minimum visual angle and VA. It will focus on early attempts to test VA, the emerging need for reliable vision tests in 19th century ophthalmology and the first optotypes around 1850. The most important improvements of these optotypes are discussed next, ignoring numerous later modifications of the first examples, ending with some recommendations and conclusions. It will disregard stereoscopic acuity, Vernier hyperacuity [7], low-contrast optotypes, spatial frequencies or integrations, cortical visual functions [8] and ultra-low vision [9].

## Thoughts on vision, visual acuity, and minimum visual angle from the ancients to the seventeenth century

About 2000 BCE, the Egyptians believed that one was reincarnated in a decan star,^1^ 70 days after death. These astronomers would have needed the equivalent of Snellen VA 30/20 to see those stars with the naked eye [10]. The Persians used a test in which each of the double stars Alcor and Mizar, separated by 11.8 min of arc (arcmin) should be seen. This test, which later was named The Arab Eye Test, would have required the equivalent of 20/20 Snellen VA [11]. The ancient Greeks had recipes for improving VA, amongst them were roasted (eyes of) eagles, owls, vultures, and frogs [12] but had no numerical evaluation of VA. Aristotle, around 350 BCE, divided VA in “average”, seeing distant objects clearly and in detail, below average and above average. He thought that VA was best in eyes with average water levels, while his predecessor Anaxagoras stated that the larger the eye, the higher the VA [12].

**Fig. 1:**
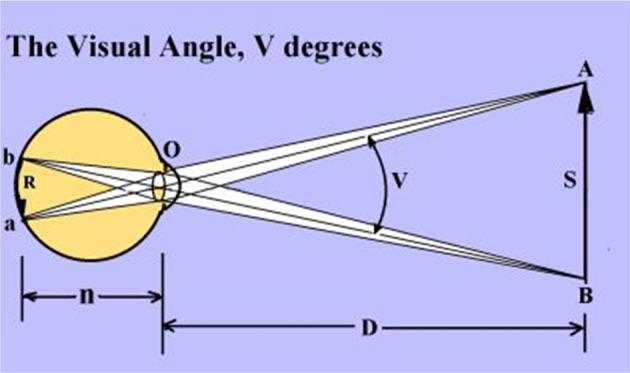
The visual angle and its minimums. The visual angle V is the angle the chief rays from object AB subtend at the nodal point O of the eye, usually expressed in degrees, minutes or seconds of arc. The central one of the three lines from A and B to O is the chief ray. The nodal point O of the eye lies about in the middle of the lens. The minimum visual angle is the smallest angle under which a stimulus S can be seen. The minimum separable angle is the minimum angle under which an observer can see two adjacent high-contrast stimuli. Visual acuity, measured with optotypes, is expressed as the reciprocal of the smallest visual angle in minutes of arc at which two objects are seen separately.

The Greeks had no documented knowledge about ophthalmic optics but they did have two theories about the seeing mechanism: The emanating or emission theory in which the eye sends rays towards objects to be seen and the immission or intromission concept of Aristotle and Epicure in which objects send rays towards the eye [13]. Nevertheless, Euclid, a mathematician living 300 BCE in Alexandria, postulated important axioms. He stated that the rays emanating from the eye move in straight lines, that they form a cone with its tip in the eye and its base on the visible object, and that we can only see objects on which rays fall. He tried to prove that there exists a minimum visible and a minimum separable angle (Fig. 1) [13]. Thus, Euclid correctly formulated our present view that VA depends on the smallest visual angle that fits an object in the cone of vision, with its tip on a photoreceptor in the retina. In a Latin translation AD 1350 of Euclid’s Greek book it says next to a geometric figure with points a, b and d “Sit igitur minimus angulus abd determinatus visui” (thus the minimum angle abd of vision should be determined) and that seems to be the first time that the expression “minimum angle” appeared [14]. The Arab scientists Avicenna and Alhazen disproved around AD 1000 the Greek emission theory [15].

## Early visual testing

It was not until the early 17th century that Hooke took up Euclid’s idea of the minimum angle again [16]. Smith [17], Jurin [18] and Duke Elder [19] attributed to Hooke using stars to test that the sharpest eye cannot distinguish the interval between two stars that are less than 0.5 arcmin apart. Hooke, however, made marks on a ruler that, at a certain distance from the eye, covered an angle of 1 arcmin. Hooke wrote *“we cannot by the naked eye make any astronomical or other observation to a greater exactness than that of a minute* [16, 20]*. And so if there be 2 or 3, or 10 or 100 small Stars so near together as that they are all comprised within the Angle of one Minute, the Eye has a Sensation of them all, as if they were one Star, and distinguishes them not one from another; so likewise is it, that the Light be strong and powerful so as to affect the Eye, It always appears of the Bigness of a Minute, though possibly its real Angle be not a second*.” [16] Later investigations confirmed Hooke’s calculations on the minimum angle [21].

**Fig. 2:**
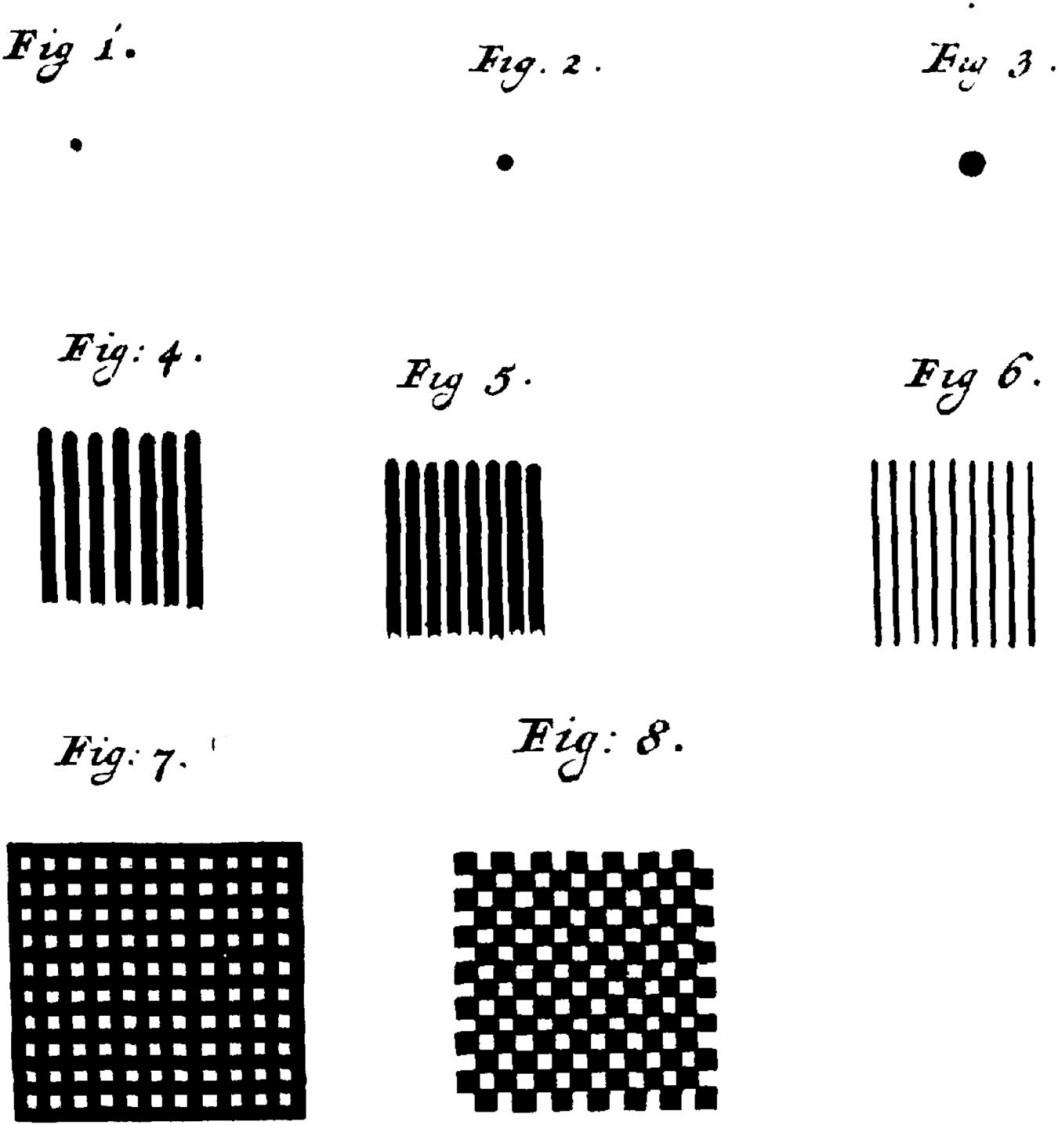
Tobias Mayer’s images for his visual acuity tests in 1754 [25]. Figures 1–8 (from the original manuscript) show various shapes, drawn on paper with Chinese ink, to investigate the limits of VA.

The first reading tests [22] and optotypes have been erroneously attributed to Benito Daça de Valdes [23]. In 1623, De Valdes instructed nearsighted customers to use a stick to measure the distance between their eye and the furthest, sharply seen mustard seed in a row on the table. By placing this stick on a picture in his book, the power of the lens needed to see in the distance could be read. This ingenious method used mustard seeds not as an object of a standardized size, seen under a specified visual angle as we would expect from an optotype, but as a convenient tiny marker to determine the far point. In 1746, Camper mentioned the influence of weather conditions on distant vision of objects under the same angle [24]. Tobias Mayer performed experiments with various test objects such as dots, lines, and grids drawn on very white paper (Fig. 2) to determine the limits of VA, that he called “terminus visionis” [25]. He expressed the VA in seconds of arc (arcsec) and studied it under different lighting and clustering conditions. He came up with a formula for primary VA using isolated test objects and secondary VA for clustered ones. Was he anticipating what would later be called visual crowding? [26] Stampfer found in 1834, with plates showing progressively thinner and closer together lines, as smallest visual angle for normal eyes 1.5 arcmin [27]. Stampfer’s images later appeared in Edward Jaeger’s first optotype book [28]. A complicating factor in creating standardized optotypes was that in Stampfer’s time every major European city had its own foot as a measure of length and even in a single city guilds (e.g. carpenters, cloth merchants, tanners) used their own inch length [29].

## Optotypes

### First attempts at visual acuity testing by optotypes

**Fig. 3:**
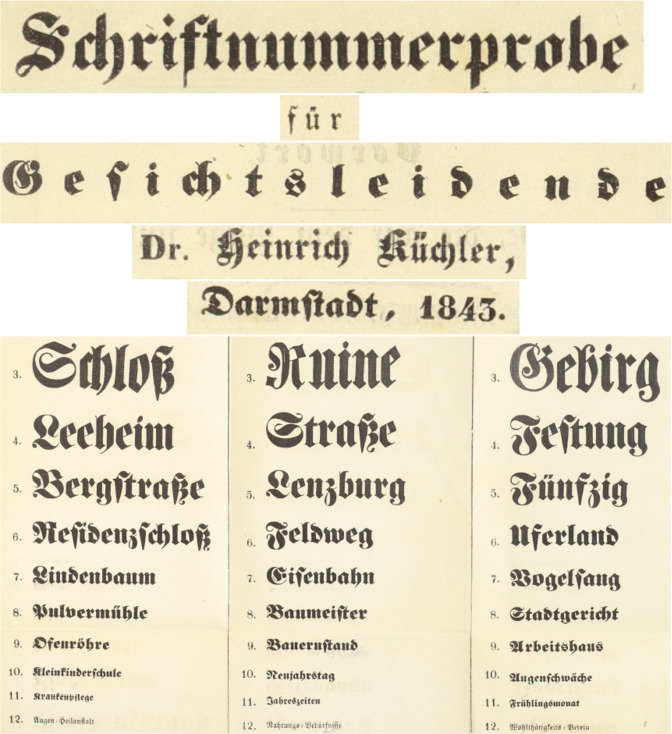
World’s first optotypes with variable font sizes by Heinrich Küchler, 1843 [38]. His instructions for their use contained excellent preconditions that optotypes must meet that are still valid today.

Opticians realized earlier than ophthalmologists, that texts and their distance from the eyes were important for prescribing good glasses [30–33]. These authors sometimes used a standard text [30] or well-known books, such as the Encyclopédie [32], with the advice to measure the letter height [31] or use a piece of string to keep the correct distance from the eye [32]. Around 1835, the German ophthalmologist Küchler cut out figures of decreasing size from almanacs and pasted them on cardboard. He soon discovered that e.g. dolls were easier to recognize than guns [34]. The next attempt at a more exact VA determination appeared in 1838 in a case report in which the patient could read Cicero print (4.2 mm) at a distance between 5 inches and 3 feet [35]. Küchler complained in 1844 that even in a file of a single patient, differently sized objects were mentioned to record changes in his VA [36]. A German textbook described in 1843 printed texts, points, crosses, or digits drawn on paper or a blackboard, and colored paper strips or discs for testing VA [37]. Küchler was sentenced in 1836 to 3 years in jail for participating in political riots [34]. After his release, he continued his ophthalmic practice and he might have invented in 1843 the world’s first optotypes with different font sizes (Fig. 3) in prison [38]. The accompanying manual included instructions (1) to print the types on white paper smoothly pasted on cardboard, (2) to start testing the poorer eye in daylight falling from the side, and (3) to note essential deviations from the weather, light or temporary body conditions, because of their influence on VA [38]. Küchler’s more remarkable insights on optotypes were (a) One should be careful not to confuse the eyesight with refractive power. (b) Choroiditis was one of the eye conditions for which optotypes helped to make a diagnosis (this was before the invention of the ophthalmoscope or slitlamp). (c) There is a large variety of test objects but not every doctor can choose his own object. One would wish that doctors of big nations, and if possible, the whole world would agree on a single test object. (d) For such an object there exist three major conditions. (1) Everybody should be able to understand the test object. (2) The test object should have no other differences but its size and the visual angle under which its extreme rays fall on the lens of the patient. (3) The subsequent objects should have as equal as possible a difference in size compared to the previous and next ones [36].

**Fig. 4:**
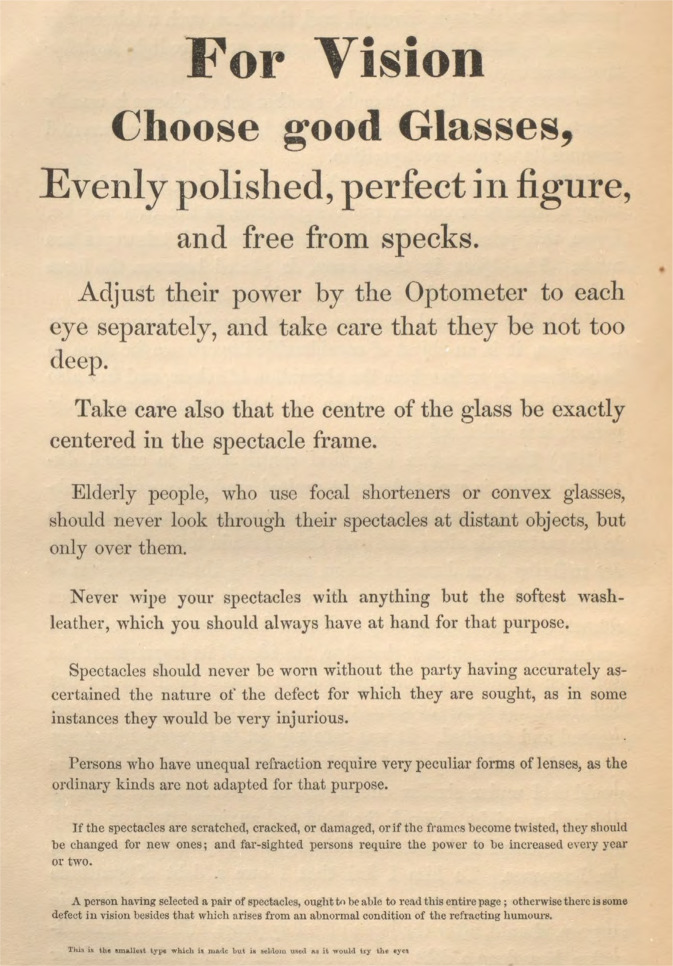
First English reading text with different font sizes [39]. In this book by Smee from 1847, no name of the test, reference to its purpose, numbering of lines, nor instructions for use were given. Possibly it was a disguised advertisement for his optometer.

**Fig. 5:**
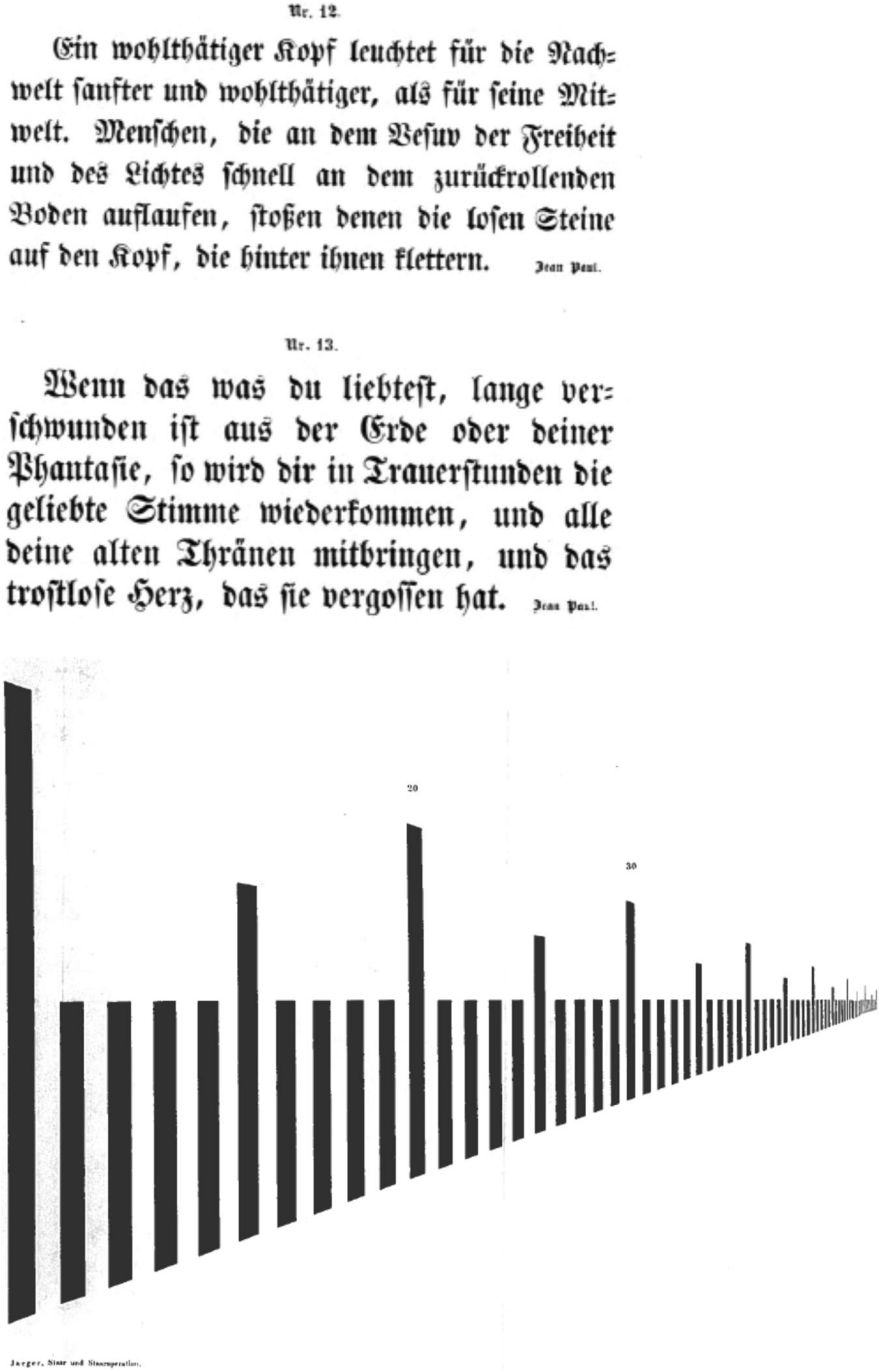
First edition of Jaeger’s reading tests, 1854 [28]. The plate with bars was provided by Stampfer [27].

**Fig. 6:**
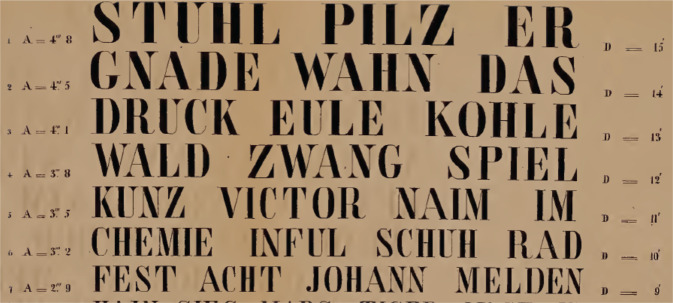
Optotypes by Stellwag von Carion 1855 [42]. A indicates the size of the letters, D the distance at which they should be read.

In 1847, the surgeon Smee published the first English reading test with different font sizes. (Fig. 4) [39]. A few years later, Eduard Jaeger’s first edition of his optotypes (Fig. 5) appeared as an attachment to a book on cataract surgery [28]. He did so in order to prove the benefits of cataract extraction. He used texts in Gothic German, English and French and four plates with progressively thinner and closer bars provided by Stampfer. His 2^nd^ edition, named Schrift-Scalen, had over 60 pages. Each page had 14 (smallest print) to 3 lines of text in 10 languages, taken from writers such as Goethe and Schiller [40]. The Stampfer bars were left out, being too difficult and time-consuming to count them reliably in practice. Jaeger gave in both early editions no instructions how to use these texts or at what distance, apart from finding the smallest print that could be read “with moderate fluency.” So they would not meet the Introduction’s definition of optotypes. The Schrift-Scalen editions printed in Austria were consistent in font size, as opposed to UK or USA releases [41] to which the advice was added at an unknown time to have them read at 14 or 16 inches. Stellwag von Carion, in 1855, issued optotypes including the distance at which each line should be read in its entirety (Fig. 6) [42]. Donders wrote that, following Albrecht von Graefe, he expressed VA loss as accurately as possible in fractions. So VA ½ or ¼ meant that the retinal image had to be two or four times larger than normal to distinguish the same shape [43]. Donders did not write how he calculated those retinal image sizes. He asked Herman Snellen, his assistant and PhD student, to find out how best to make a reliable test for VA [44]. It is often assumed that Snellen’s optotypes were invented by Donders, but Donders explicitly wrote in a footnote: “*Dr. Snellen heeft een systeem van dergelijke letters ontworpen, gaande van CC tot I, zeer geschikt, om den graad der gezichtsscherpte te bepalen.”* (Dr. Snellen developed a system of such letters, ranging from CC to I, very suitable for determining the degree of VA) [45] and later repeated this more extensively [46]. Snellen studied the refraction of thousands of patients and this resulted in his first optotype edition in 1862 [4].

### Accommodation and visual acuity testing

Until Donders’ book *On the anomalies of accommodation and refraction of the eye* [46], ophthalmologists and optometrists did not know that shortsighted and hyperopic people could also accommodate. Snellen, who later coined the word eidoptometry for VA testing [47], still wrote that VA is only perfect on condition of maximal accommodation for the given distance. Later it became clear that accommodation should be eliminated as much as possible when determining VA with the best correcting refraction [46].

### Average visual acuity and early reflections on optotypes

**Fig. 7:**
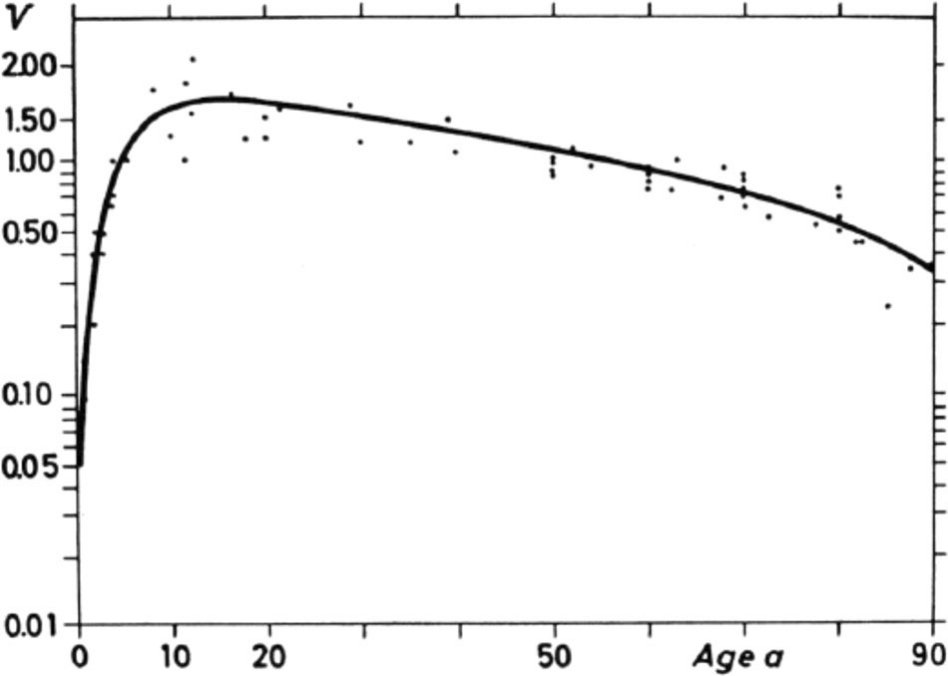
Mean visual acuity V versus age in years per decade. Each dot represents the result of one study [48].

**Fig. 8:**
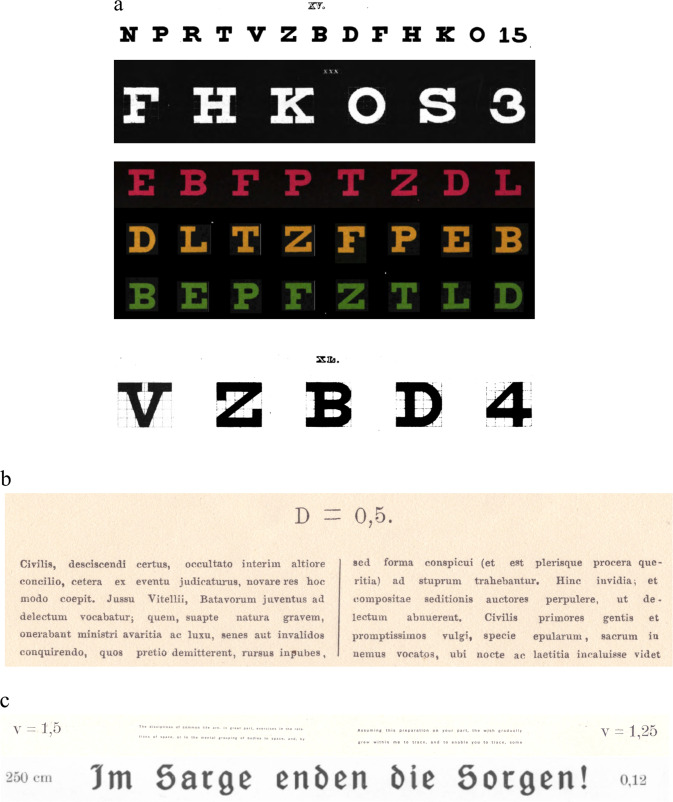
Optotypes by Snellen and Birkhäuser. **a** The roman number above the letter row indicates the distance D in Paris feet (after 1875 in meters) at which the letter subtends a visual angle of 5 arcmin [4]. **b** Smallest font size (*D* = 0.5) in Snellen’s international reading test, 1875 [5]. In this Latin text, Snellen used the word optotypi for the first time. **c** Smallest font size in Birkhäuser’s reading test at 30 cm, *V* = 1.25 [67]. This barely legible, curious text of *V* = 0.12 reads: *Im Sarge enden die Sorgen!* (Worries cease in the coffin!).

It is sobering to read what Snellen wrote about the visible minimum. *“The angle of 5 arcmin which Snellen’s test types are based on, is arbitrarily assumed. It does not give the average VA. This cannot be determined with certainty at all because it is influenced by many moments. The angle of 5 arcmin is approximately the mean VA, if one also counts older eyes in a statistical examination. It constitutes by no means the maximum of normal vision, in that many, especially young eyes have greater VA*,” [47] as was later confirmed (Fig. 7) [48]. Snellen wrote that VA is inverse to the number of photoreceptors in the retina covered by the smallest angle under which a certain image can be perceived, but also that VA is the inverse of this smallest visual angle, and thus is not an absolute but a relative value. He pointed out that some letters were more often misspelled than others [47] Snellen noted that the criterion for seeing is “clear seeing, not unclear recognition” but did not mention how to distinguish these two. In addition, he commented on many other aspects while testing VA like pupillary diameter, light levels just prior to testing, white letters on a black background having better visibility for cases with poor VA, and on reading not being the same as recognizing individual letters. This has recently been experimentally confirmed [49]. Reading speed is important and was hampered by visual field loss [4]. Snellen also stated that the letters in Jaeger’s Schrift-Scalen of 1859 were not square and unequal in thickness, height and clarity of printing [4]. Snellen seems to have been the first to design letters based on a similar visual angle of 5 arcmin with 1 arcmin for the letter legs. He approximated, without explicitly mentioning it, geometric progression (multiplication by a constant factor) in font size for subsequent lines, each line having letters 1.25–1.5 times larger than the previous one. In his earlier optotype editions (Fig. 8), Snellen expressed the distance between the patient and the letters in Paris feet, 20 feet being equal to about 6.5 m. From 1875 on, he embraced the meter unit.

In 1864, the British military medical service ordered 1000 of Snellen’s test types [50] and the distribution of these over the world certainly contributed to their later popularity. Professional jealousy arose between Jaeger and Snellen [41]. Snellen tried in vain to convince Jaeger that he should specify the distance for reading his texts and that he should use the minimum visual angle [41]. Twelve years after his first optotype booklet, Snellen wrote: *“Retinal perception and VA should be separated from each other. In order to express the viewing angle in easily comparable dimensions, one has to base its determination on a conventional unit.”* This is now fairly generally assumed to be an angle of 5 arcmin in order to recognize letters whose thickness is 1/5 of their height. The distance d at which such letters can still be clearly recognized divided by the distance D whereupon they appear at an angle of 5 arcmin then expresses the VA, *V* = *d*/*D* [47]. The discussion about VA testing and whether 5 arcmin was the best unit for an optotype, has long continued at international ophthalmological congresses [51–53]. Vierordt [54] challenged Snellen and wrote that the square of the diameter of the retinal image should be in his denominator and not its simple diameter. When optotype 20 is recognized at 20 feet, in his view VA = 1. However, at 10 or 5 feet, the VA should not be 10/20 (1/2) or 5/20 (1/4) as Snellen wrote, but 10/40 and 5/40 [54]. Snellen and Donders evaded the essence of this criticism and wrote that they preferred this simpler way of expression, because everywhere with all optical instruments the size of the images is expressed in linear dimensions [47].

### Towards standardization of test charts

**Fig. 9:**
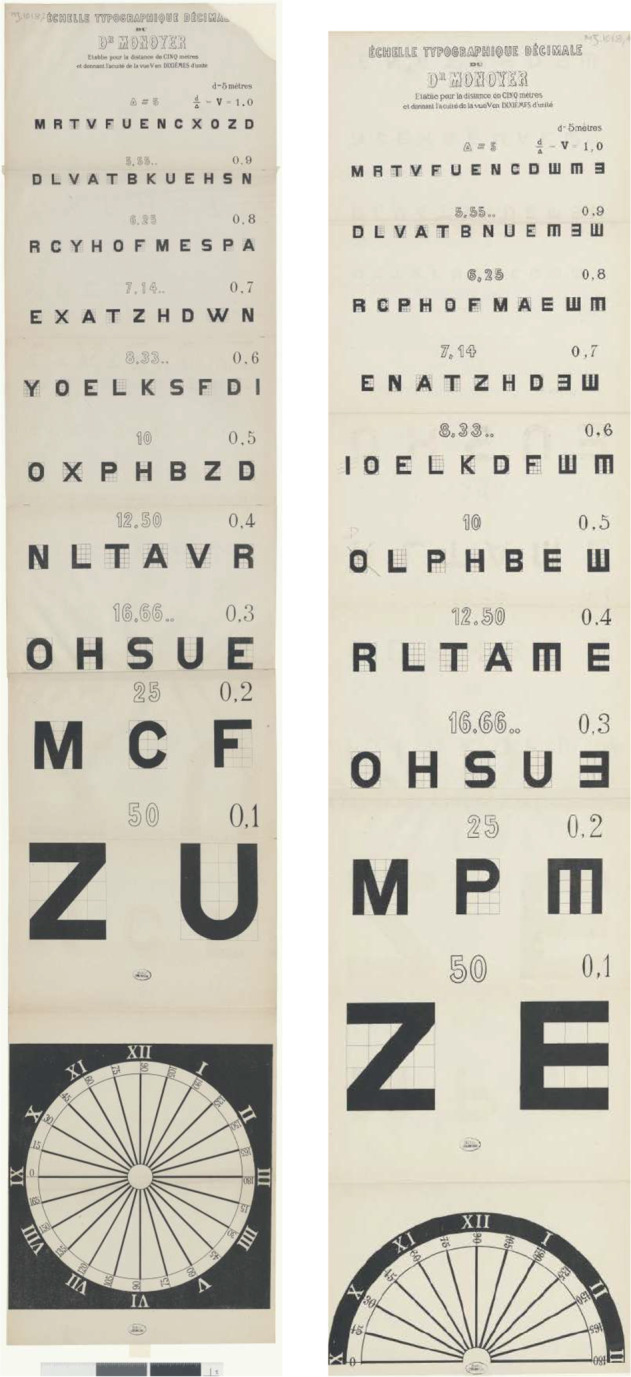
The first decimal optotypes by Monoyer 1875 [55]. From bottom to top above the ZU chart one can read on the left MONOYER and on the right FERDINAND.

**Fig. 10:**
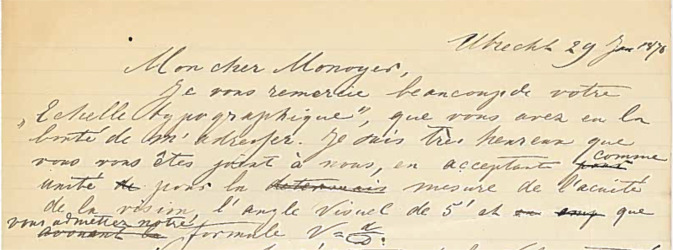
Concept letter of Herman Snellen Sr to Ferdinand Monoyer (Utrecht 29 June 1876) [68]. My dear Monoyer, I thank you very much for your typographic scale which you were kind enough to send me. I am very happy that you have joined us in accepting as the unit for measuring the acuity of vision, the visual angle of 5′ and that you accept our formula *V* = *d*/*D*.

**Fig. 11:**
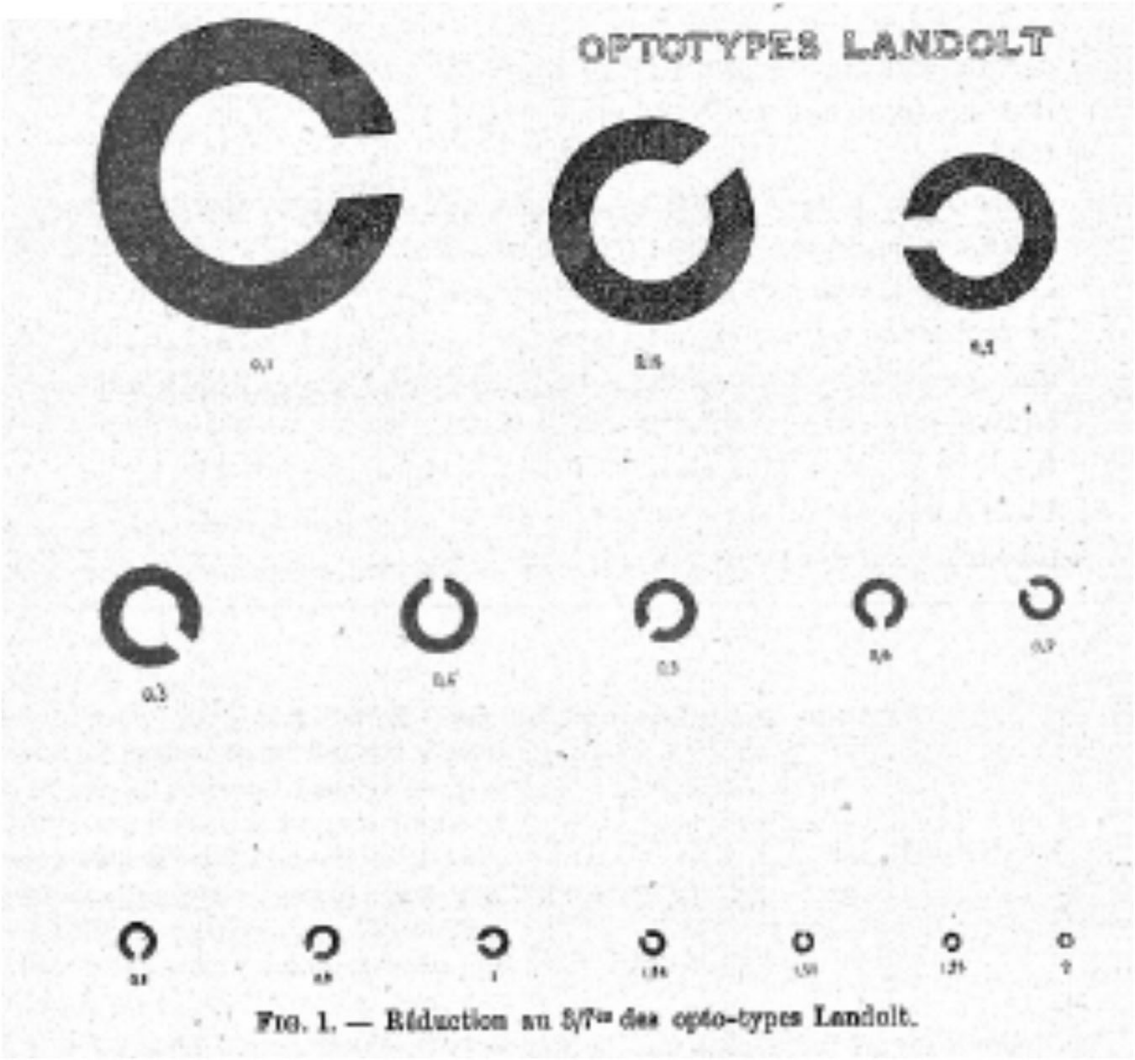
Landolt’s C optotypes [56]. The opening in the C can be in eight places, so naming this position is a forced-choice task with eight alternatives [65].

After Jaeger and Snellen, dozens of optotypes appeared instigated by improvement, fame or gain (Table 1). Some of the more important ones are discussed here. In 1876, Monoyer issued his decimal test card with letters without serifs using distances in meter (Fig. 9) [55]. Monoyer sent Snellen a complimentary copy for which the latter thanked him, expressing his joy that Monoyer accepted the angle of 5 arcmin as the unit for optotype size, as well as Snellen’s formula *V* = *d*/*D* (Fig. 10). The progression of font size of the optotypes can be geometrical or arithmetical (constant difference between two types). Monoyer’s chart was arithmetical. Landolt’s C chart was the first to fulfill three of Küchler’s conditions (Fig. 11) [56]. Psychophysicists like Louise Sloan realized that the letter Z was correctly read in 94% of tested eyes and the S only in 70% [57]. A subcommittee of the American Medical Association selected 10 letters of medium difficulty, used Snellen’s principle that the height and width of a letter should be 5 times its leg width, and chose 0.1 log unit as the geometrical magnification factor, resulting in the Sloan chart [57].

**Table 1:** Chronological arrangement of earliest editions of major reading tests or optotypes.

Year		Ref.
1834	Stampfer	[27]
1843	Küchler	[38]
1847	Smee	[39]
1854	Jaeger von Jaxtthal	[28]
1855	Stellwag von Carion	[42]
1862	Snellen	[4]
1862	Giraud-Teulon	[69]
1868	Green	[70]
1869	Burchardt	[71]
????	Hardy	[72]
1874	Galezowski	[73]
1875	Monoyer	[55]
1883	Nieden	[74]
1884	Pflüger	[75]
1885	Dennett	[76]
1885	Olivier	[77]
1888	Parinaud	[78]
1899	Landolt	[56]
1905	Bjerke	[79]
1906	Koster	[80]
1911	Birkhäuser	[67]
1929	Beach	[81]
1951	Law	[82]
1958	Keeney	[83]
1959	Sloan	[57]
1976	Bailey	[59]
1982	Ferris	[60]
1988	Colenbrander	[84]
1998	Radner	[64]
2017	Alabdulkader	[66]
2020	Ayton	[9]

A few years later Bennett, the chairman of the British Standards Institution Sub-Committee on Ophthalmic Test Types wrote “The many attempts to improve on Snellen’s work have led to such divergences that existing charts no longer provide a comparable basis for estimating visual acuity.” [58] As principal causes for these deficiencies he mentioned a difference of opinion on three fundamental questions, a. The style and selection of the optotypes, b. The progression of sizes between them and c. The notation for recording VA. The VA can be expressed in an absolute or a relative value. Absolute would be the threshold visual angle expressed in arcmin, thus returning to Mayer [25]. The relative method is similar to the Snellen notation *V* = d/D, in which the VA is expressed against a standard D: the distance to the letter subtending 5 arcmin.

### Introduction of LogMAR charts

**Fig. 12:**
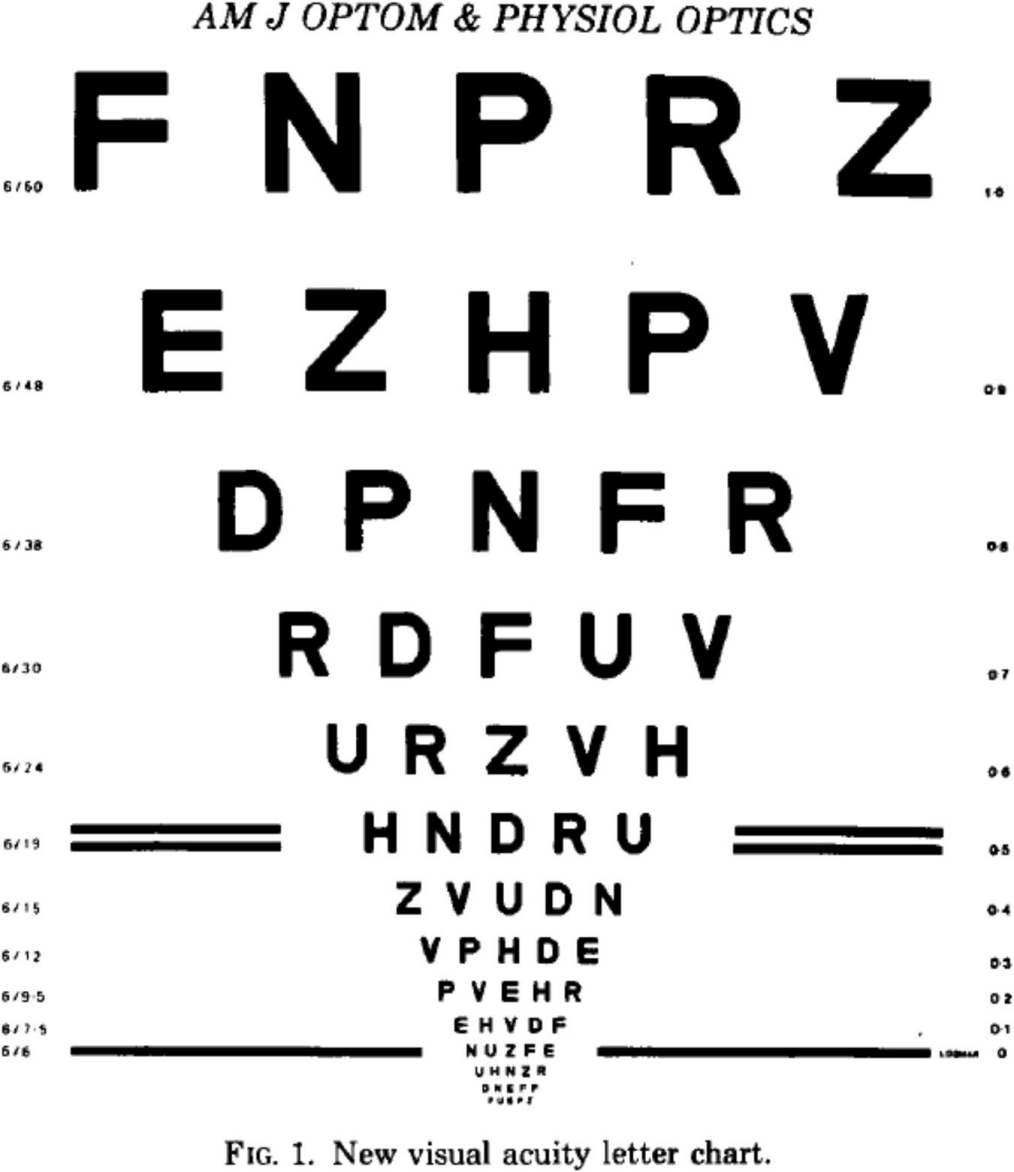
The first LogMAR chart by Bailey and Lovie from 1976 [59]. This chart formed the basis for the mostly used optotype today, the ETDRS chart.

Bennett outlined the problems but did not really come up with solutions and left these to Bailey and Lovie [59]. These authors were able to dispel several of Bennett’s criticisms by using five letters of equal legibility on each row, uniform spacing between letters and rows, a 0.1 log unit progression of font size, and the possibility of letter by letter scoring (Fig. 12). By using a standard viewing distance of 6 meters, they noted the VA as the Logarithm of the Minimal Angle of Resolution and thus the LogMAR chart was created. Counter-intuitively to users of Snellen charts, its lowest VA was 1.0 and its highest -0.3 and that may be one of the reasons why the LogMAR VA notation is the standard in research today but not in the more conservative clinics. Since Bailey and Lovies’s chart, over 10 new LogMAR charts have been developed of which the ETDRS one became best known [60]. The same lament that Bennett uttered about the Snellen types, held for some of these LogMAR charts [61]. The number of optotypes per row varied from 4 to 10, the height and width of optotypes from 4 to 6.4 arcmin, space between rows from 36 to 56 arcmin, and spacing between the optotypes was irregular [61].

### Reflections after 170 years of optotyping

In retrospect, we see that precursors of optotypes originated with the opticians, that ophthalmologists refined them, and that their improvements came from visual scientists. The fruits of these disciplines became more diverse, partly due to differing emphasis on the accuracy of VA determination as workload in clinics competed with exactitude. See for example, the disappearing Stampfer bars in Jaeger’s Schrift-Scalen and the replacement of several lines of Snellen letters by a single row because of “*very considerable inconvenience, especially with dull patients, and in the hurry of hospital and infirmary practice*”. [62] Also the impossibly small reading test in daily practice (Fig. 8c). Mayer’s two types of VA for single and crowded test objects could be reduced to one [25]. Küchler’s VA variations due to different lighting were solved, but not those caused by atmospheric or patient’s health changes [38]. How a test should be administered or assessed was extensively researched [9, 49, 63]. One tackled the lack of clarity from Jaeger’s reading tests, checking reading ability “with moderate fluency,” and Snellen’s “clear seeing, not unclear recognition,” to how an examiner should judge the result of a VA test, by measuring reading accuracy and speed [64]. For best-corrected VA, the refraction has to be accurately determined beforehand with optotypes, so that Snellen’s “maximal accommodation for the given distance” was eliminated. Snellen mentioned several variables in testing, among others the difference between letter recognition and reading [4] which was emphasized again 150 years later [49, 64]. The LogMAR charts solved problems of easy and difficult letters and whether all optotypes on a line have to be read flawlessly or not. LogMAR charts, however, have different alternative forced-choice formats and termination rules, depending on allowance to read a whole line or to stop after a specific number of errors. A cut-off criteria of four mistakes on a line seems best for Bailey-Lovie and ETDRS charts [65]. Starting a VA test with the poorer eye was not necessary anymore due to separate charts for each eye and these also prevented remembering optotypes from the fellow eye examination [60]. There are several continuous-text reading tests including one registering reading speed, solving the dilemma how much time will be allowed to complete a test [9, 63]. Only a limited number of modern reading tests use a string to maintain the correct distance from the eyes to the text. VA testing can still be hampered by unknown illiteracy, dyslexia or cognition problems. The wide variation in alphabets around the world [63, 66] makes it unlikely that 150 years of research on Roman letters has been applied to other alphabets and even Arabic numerals cannot be read worldwide. We still are a long way from Küchler’s pursuit of international, standardized, and reliable optotypes, understandable to everyone. Remarkable, when one considers the consequences of VA in assessing whether someone is allowed to drive, is eligible for social benefits or is able to go to the Olympic or Paralympic games. Over 100 research groups around the world, involved in developing new therapies for (nearly) blind persons, are now collaborating in the HOVER task force to develop new VA tests and criteria for ultra-low vision [9].

## Conclusions

A first step towards global unified VA testing would be to apply all the basic rules of correct LogMAR charts to other alphabets or, preferably, to use simple optotypes such as tumbling C or E types. Had the creators of the Arab Eye Test found more stars of different magnitudes and distances in the entire universe, Küchler’s ideal of global optotypes could have been obtained thousands of years ago at night skies without light pollution. Perhaps the sun and the moon should now have been added to these stars for persons with ultra-low vision but unfortunately, “the sky is the limit” would have a different meaning for them.

## Footnotes

1 A decan star rose at the horizon just before sunrise at the beginning of a decade and thus the year had 36 groups of decan stars, 360 days. The Greek astronomer Hipparchus introduced 150 BCE a classification for magnitudes (brightness) of stars from 1 (brightest) to 6. Some 70 decan stars of magnitude 1–5, associated with Egyptian reincarnation had names and these were often depicted on the lids of mummy boxes and in tombs.
