# Correction to: A history of visual acuity testing and optotypes

**DOI:** 10.1038/s41433-023-02612-x

**Published:** 2023-07-05

**Authors:** Paulus T. V. M. de Jong

**Affiliations:** 1 Department of Retinal Signal Processing, Netherlands Institute of Neuroscience, KNAW. Meibergdreef 47, 1105 BA Amsterdam, The Netherlands; 2 Department of Ophthalmology, AmsterdamUMC, Meibergdreef 9, 1105 AZ Amsterdam, The Netherlands

**Keywords:** Scientific community, Diagnosis

Correction to: *Eye* 10.1038/s41433-022-02180-6, published online 03 August 2022

Due to a typesetting mistake, the sentence “A decan star rose at the horizon just before sunrise at the beginning of a decade and thus the year had 36 groups of decan stars, 360 days. The Greek astronomer Hipparchus introduced 150 BCE a classification for magnitudes (brightness) of stars from 1 (brightest) to 6. Some 70 decan stars of magnitude 1–5, associated with Egyptian reincarnation had names and these were often depicted on the lids of mummy boxes and in tombs.” was originally not typeset as a footnote. Additionally, in the caption of Figure 1, the explanation of visual acuity was added: “Visual acuity, measured with optotypes, is expressed as the reciprocal of the smallest visual angle in minutes of arc at which two objects are seen separately.” The caption title of Fig. 8 was also corrected to read “Optotypes by Snellen and Birkhäuser”. The original article has been corrected.

